# Global 5-hydroxymethylcytosine content is significantly reduced in tissue stem/progenitor cell compartments and in human cancers

**DOI:** 10.18632/oncotarget.316

**Published:** 2011-09-02

**Authors:** Michael C. Haffner, Alcides Chaux, Alan K. Meeker, David M. Esopi, Jonathan Gerber, Laxmi G. Pellakuru, Antoun Toubaji, Pedram Argani, Christine Iacobuzio-Donahue, William G. Nelson, George J. Netto, Angelo M. De Marzo, Srinivasan Yegnasubramanian

**Affiliations:** ^1^Sidney Kimmel Comprehensive Cancer Center, Johns Hopkins University, Baltimore, Maryland, USA; ^2^Department of Pathology, Johns Hopkins University, Baltimore, Maryland, USA; ^3^Brady Urological Institute, Johns Hopkins University, Baltimore, Maryland, USA; ^4^Department of Medicine, Division of Hematology, School of Medicine, Johns Hopkins University, Baltimore, Maryland, USA

**Keywords:** 5-hydroxymethylcytosine, 5hmC, DNA methylation, differentiation, cancer, tissue stem/progenitor cells

## Abstract

DNA methylation at the 5-position of cytosines (5mC) represents an important epigenetic modification involved in tissue differentiation and is frequently altered in cancer. Recent evidence suggests that 5mC can be converted to 5-hydroxymethylcytosine (5hmC) in an enzymatic process involving members of the TET protein family. Such 5hmC modifications are known to be prevalent in DNA of embryonic stem cells and in the brain, but the distribution of 5hmC in the majority of embryonic and adult tissues has not been rigorously explored. Here, we describe an immunohistochemical detection method for 5hmC and the application of this technique to study the distribution of 5hmC in a large set of mouse and human tissues. We found that 5hmC was abundant in the majority of embryonic and adult tissues. Additionally, the level of 5hmC closely tracked with the differentiation state of cells in hierarchically organized tissues. The highest 5hmC levels were observed in terminally differentiated cells, while less differentiated tissue stem/progenitor cell compartments had very low 5hmC levels. Furthermore, 5hmC levels were profoundly reduced in carcinoma of the prostate, breast and colon compared to normal tissues. Our findings suggest a distinct role for 5hmC in tissue differentiation, and provide evidence for its large-scale loss in cancers.

## INTRODUCTION

Epigenetic modifications play a crucial role in cellular differentiation and have been implicated in numerous disease states including cancer [1-4]. One of the most studied of these modifications is the addition of a methyl group on the 5-position of the cytosine (5mC) base in a CpG dinucleotide. Accumulation of methylation marks in CpG rich regions around the transcriptional start site of genes has been show to be associated with alterations in chromatin organization ultimately leading to changes in locus specific transcriptional activity [1]. Paradoxically, DNA methylation marks can be heritably maintained across cell division but can also be reversibly/dynamically altered to establish new epigenetic programs. However, major uncertainties remain on how cells can erase existing methylation marks [2, 5, 6].

The recent discovery of a group of enzymes of the ten-eleven translocated (TET) family that can specifically modify these DNA methylation marks by oxidizing 5-methylcytosine (5mC) to 5-hydroxymethylcytosine (5hmC) has added another dimension of complexity to our understanding of DNA methylation [7]. It has been well established for decades that certain bacteriophages contain 5-hydroxymethylcytosine rather than cytosine in their genome to protect themselves from host-controlled nucleases [8]. The presence of 5hmC in mammalian cells has historically been very controversial, and its role in mammalian genomes is not well understood [9-11]. Interestingly, Penn et al. demonstrated in 1972 that 5hmC can be detected by crude chromatography methods in rodent brain and liver DNA preparations [11]. More recently, using mass spectrometry, Kriaucionis and Heintz provided firm evidence for the presence of 5hmC in Purkinje cells of the murine cerebellum [12]. Subsequently, several studies have addressed the potential role of 5hmC and the oxidizing enzymes of the TET protein family in genome organization and differentiation of murine embryonic stem (ES) cells [13-21]. The tissue specific cellular distribution of 5hmC in normal adult tissues and neoplasia, however, has thus far not been well-documented.

To address these questions, we applied a novel immunohistochemical staining method to detect 5hmC in a variety of normal murine and human tissues. Interestingly, we found that in embryonic and adult tissues, the abundance of 5hmC correlates with cellular differentiation, with more differentiated cells showing higher 5hmC staining. Furthermore, we observed an almost uniform loss of 5hmC levels in cancer tissues as compared to their normal counterparts, suggesting a complex and yet to be defined role of 5hmC in tissue differentiation and neoplasia.

## RESULTS

### Development and validation of an immunohistochemical staining method for global analysis of 5hmC levels *in situ*

**Figure 1:**
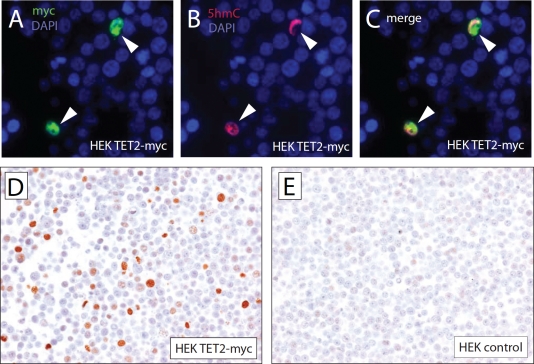
Specificity of immuohistochemical detection of 5hmC To assess the specificity of 5hmC immuno-labeling of formalin fixed paraffin embedded cells, HEK293 cells were transfected with expression plasmids encoding for myc-tagged TET2 or control. Cell pellets were fixed and embedded in paraffin. Sections of the resulting paraffin block were co-immuno-labeled with anti-myc and anti-5hmC specific antibodies and visualized using fluorophore conjugated secondary antibodies (A, B, C). Note that only cells that express high levels of TET2 (indicated by arrowheads) showed strong staining for 5hmC. (E, F) To show that 5hmC can be specifically detected using a chromogenic immunohistochemistry method, HEK293 cells overexpressing TET2-myc and HEK293 control cells were stained with 5hmC specific antibodies and immunocomplexes were visualized using HRP conjugated secondary antibodies with DAB as a chromogen.

Investigation of tissue-specific 5hmC distribution has so far been attempted by using quantitative mass-spectrometry based methods or semi-quantitative antibody-based immunofluorescence microscopy [12, 22]. Unfortunately, global 5hmC detection methods involving processing of tissue lysates do not allow the evaluation of 5hmC levels on a cell-by-cell basis. Immunofluorescence microscopy, on the other hand, does not allow full morphological evaluation of the tissue and is often confounded by auto-fluorescence background, complicating interpretation. We therefore aimed to develop a method that allows the immunolabeling of 5hmC with a commercially available and recently extensively validated polyclonal antibody [13, 22] and subsequent immunohistochemical detection. To evaluate the specificity of the antibody, HEK293 cells were transiently transfected with expression vectors encoding myc-tagged TET2 or control (Figure 1). Cells were fixed in 10% buffered formalin and embedded in paraffin as described previously [23]. Sections of the obtained cell block were then double-immunolabeled with 5hmC and myc-tag specific antibodies. As shown previously [24], cells expressing TET2 (arrowheads) showed strong nuclear 5hmC staining providing a robust positive control for staining optimization (Figure 1 A, B, C). Conversely, HEK293 not expressing TET2myc and control HEK293 cells did not exhibit strong staining for 5hmC. Next, TET2 expressing and control HEK293 cells were incubated with 5hmC antibodies; immunocomplexes were visualized using HRP conjugated secondary antibodies with DAB as a chromogen. Cells expressing TET2 showed a strong nuclear signal for 5hmC, whereas control transfected cells only showed very faint to undetectable nuclear staining. The low intensity of staining in the control cells likely reflects the low levels of 5hmC previously observed in HEK293 cells [25]. Our pretreatment protocol included two antigen retrieval steps: a 30 min steaming in citrate buffer (ph 6.0) and a 15 min incubation in 3.5 N HCl. Both steps were required for efficient immunolabeling of 5hmC in formalin fixed paraffin embedded material; omission of the citrate steam and/or HCl steps resulted in almost complete absence of 5hmC staining (Supplementary Figure 1), highlighting the importance of adequate antigen unmasking for immunohistochemical analysis.

### Distribution of 5hmC content in mouse embryonic tissues

**Figure 2:**
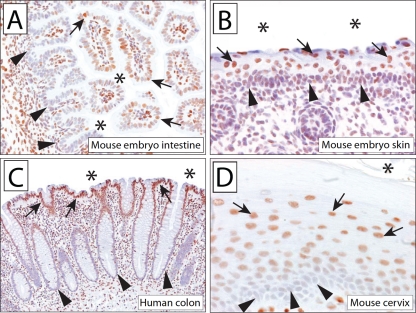
5hmC is abundant in embryonal and adult tissues, with differential abundance in basal vs. luminal cell compartments of stratified epithelia Micrographs show 5hmC staining in the intestine (A) and skin (B) of a 17 day old mouse embryo. Note the reduced staining of 5hmC staining in the basal cell compartment (indicated by arrowheads) compared to the luminal/apical epithelial cells (indicated by arrows). (C) Normal human adult colonic mucosa exhibits strong staining for 5hmC in apical epithelial cells (indicated by arrows); epithelial cells in the base of the crypt (indicated by arrowheads) show greatly reduced staining intensities. Note the strong 5hmC staining of associated stromal nuclei. (D) Hierarchical distribution of 5hmC staining in murine cervix. Asterisk (*) indicates apical/luminal surface.

We first determined the 5hmC staining pattern in the developing mouse embryo. Seventeen-day-old mouse embryos were fixed, paraffin embedded, and processed as outlined below. In line with recent reports, we detected significant levels of 5hmC in the mouse cerebral cortex and cerebellum [12, 22, 26]. In addition, 5hmC was also detectable in the majority of tissues throughout the mouse embryo. Interestingly, we observed a strong association of 5hmC content with the differentiation state of cells in many hierarchically organized tissues. For instance, in the intestine of the embryo, cells lining the crypts of the mucosa showed almost no staining for 5hmC, whereas more apical cells exhibited strong staining (Figure 2A). Similarly, the skin in the developing mouse embryo also showed a hierarchical distribution of 5hmC staining, with cells in the basal epithelial layer showing very low staining intensities and more apical cells staining strongly for 5hmC (Figure 2B). These patterns suggest that in the developing embryo, 5hmC is more abundant in more differentiated cell compartments than in the less differentiated cell compartments.

### 5hmC content is generally correlated with differentiation state of cells in hierarchically organized mouse and human adult tissues

To test whether this association of 5hmC with differentiation in hierarchically organized tissues would also be maintained in adult tissues, we investigated several tissue types from adult mice and humans. Human colon represents a classical model for hierarchical tissue differentiation. Cells at the base of the colonic crypt proliferate and represent the regenerative tissue stem/progenitor cell compartment [27, 28]. Conversely, cells in the luminal side of the colon form the terminally differentiated cell compartment. Interestingly, we found that this hierarchical differentiation is associated with strong differences in 5hmC levels. Whereas apical cells of the colonic mucosa show strong 5hmC staining, cells in the base of the crypts had greatly reduced 5hmC levels (Figure 2C). Other stratified epithelia, including that in cervix, oral mucosa, and bladder, exhibited a similar distribution of 5hmC staining in which apical cells showed higher 5hmC levels as compared to basal cells (Figure 2, Supplementary Figure 2).

**Figure 3:**
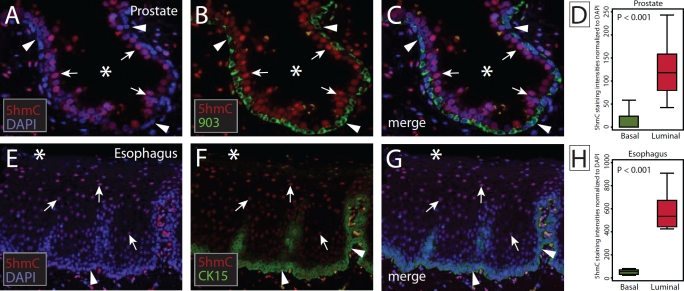
Quantitative analysis of the hierarchical distribution of 5hmC in stratified epithelia (A-C) Representative micrographs of normal prostate epithelia co-immunolabeled for 5hmC (red) and basal cell specific cytokeratin 903 (green). Nuclei were counterstained with DAPI (blue). (D) Box-plots show the distribution of 5hmC fluorescence intensities in basal (903+) and luminal (903-) cells, normalized to DAPI. (E-G) Representative micrographs of normal esophageal mucosa co-immunolabeled for 5hmC (red) and basal cell specific CK15 (green). (H) Distribution of 5hmC staining intensities in basal (CK15+) and luminal (CK15-) cells, normalized to DAPI. Arrowheads indicate basal cells, arrows indicate luminal cells, Asterisks (*) indicate lumen.

To assess this differential distribution more rigorously and quantitate 5hmC levels in luminal and basal cell compartments, we used immunofluorescence microscopy coupled with quantitative image analysis. Slides containing normal human prostate or normal human esophagus were co-immunolabeled with 5hmC antibodies and basal cell specific cytokeratin antibodies (34βE12-903 for prostate, CK15 for esophagus) [29]. Signal intensities of 5hmC were determined in basal and luminal/apical cell compartments (Figure 3) using quantitative image analysis software. We observed a statistically significant difference in 5hmC staining intensities between basal and luminal cells for prostate (median signal intensity values: basal 0, luminal 118, p < 0.0001) and esophageal epithelia (median signal intensity values: basal 53.6, luminal 555.2, p < 0.0001) providing a quantitative validation of the differential distribution of 5hmC in these tissues (Figure 3).

### 5hmC levels are reduced in hematopoietic stem and progenitor cells compared to more differentiated counterparts

**Figure 4:**
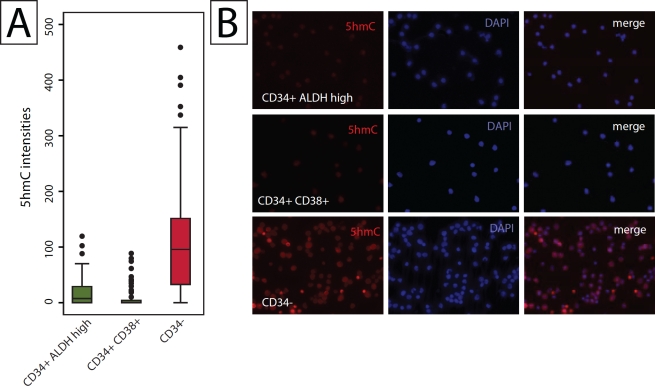
Quantitative analysis of the hierarchical distribution of 5hmC in hematopoietic cells Ficoll-Paque enriched, CD34-depleted bone marrow, or FACS sorted CD34+;CD38-;ALDH-high hematopoietic stem cells, or CD34+;CD38+ progenitor cells were spotted on glass slides, stained with 5hmC specific antibodies and visualized using immunofluorescence microscopy. Signal intensities were determined by quantitative image analysis. (A) Distribution of 5hmC signal intensities in the stem cell (CD34 positive ALDH high), progenitor cell (CD34, CD38 positive) and differentiated cell (CD34 negative) compartments. (B) Representative micrographs of each enriched fraction.

Although not necessarily hierarchically organized by location of cell compartments, hematopoietic cells in the bone marrow show a distinct hierarchy of differentiation. Well-defined markers allow the cell compartment specific enrichment of undifferentiated stem cells, progenitor cells, and terminally differentiated mature blood cells [30, 31]. Using FACS, hematopoietic stem cells (CD34+;CD38-;ALDH+) and progenitor cells (CD34+;CD38+) were sorted as described previously [32]. Cells were then stained with 5hmC specific antibodies and staining intensities in hematopoietic stem and progenitor cells were compared to more differentiated bone marrow cells that were depleted of CD34 positive cells (Figure 4, Supplementary Figure 3). Consistent with what was observed for stratified epithelial tissues as described above, hematopoietic stem and progenitor cell populations exhibited much lower 5hmC content than their more differentiated CD34 negative counterparts (Figure 4; p < 0.001).

### Loss of 5hmC in human cancers

**Figure 5:**
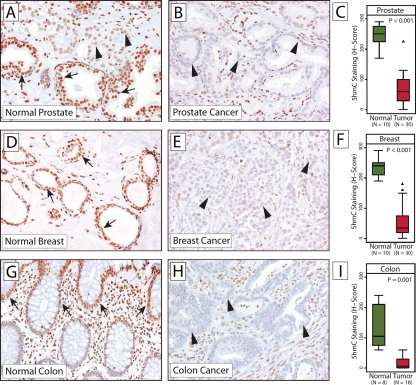
Significant reduction in 5hmC levels in cancers Micrographs of representative 5hmC staining in normal human prostate (A) and prostate adenocarcinoma (B), normal breast (D) and ductal breast cancer (E) and normal colon mucosa and adenocarcinoma of the colon (H). (C, F, I) show distributions of semi-quantitative intensities scores in normal and tumor cells in box-and-whisker plots. Note that (A) contains a small focus of cancerous glands (indicated by arrowheas) infiltrating normal prostatic epithelium. Arrows indicate normal epithelial cells; arrowheads show tumor cell nuclei with reduced 5hmC staining.

Tumors often adopt a caricaturized differentiation phenotype consisting of loss of some features of differentiation and gain of certain functions, such as self-renewal, that are more characteristic of less differentiated stem cells [33]; these changes are nearly universally associated with profound epigenetic alterations [3, 4, 34]. We assessed whether tumor cells have 5hmC contents closer to terminally differentiated cells or to tissue stem cell compartments from their tissue of origin. To determine the levels and distribution of 5hmC in cancer and normal tissues, we assessed a total of 78 carcinoma and 28 normal tissue samples from prostate, breast, and colon (Figure 5). Analysis of this set of normal tissues confirmed the general pattern of increased 5hmC content in more differentiated cell types in the normal prostate and colon; terminally differentiated luminal cells in these tissues showed much stronger 5hmC staining than basal cells, the likely compartment containing the tissue stem/progenitor cells [27, 28, 35, 36] (Figure 5A,G). In breast tissue, the identity of the undifferentiated tissue stem cell compartment is more controversial [37]. We observed that the myoepithelial cells in normal breast glands tended to show a subtle, but noticeable, stronger 5hmC staining than the normal luminal cells. Nonetheless, comparing these normal tissues to cancers arising from the same tissues, we observed a profound reduction in 5hmC content in the cancers for all three tumor types (p < 0.001 for prostate and breast; p = 0.001 for colon). Interestingly, in prostate tissues, where we could observe normal prostate glands adjacent to malignant glands, we saw a significant reduction in 5hmC staining in the cancerous glands compared to the adjacent normal glands (Figure 5A arrowheads). 5hmC staining intensities were not associated with clinicopathological features such as grade and stage. Even small lesions of low histological grade showed profound reduction of 5hmC. This suggests that the global loss of 5hmC could be an early event in carcinogenesis.

Since 5mC is the substrate for the TET-enzyme mediated conversion to 5hmC, the global loss of 5hmC seen here could simply reflect a decrease in 5mC levels, which is known to occur in human cancers. Therefore, we assessed 5mC levels in normal and tumor tissues from the colon and the prostate using a previously validated immunohistochemical staining method that specifically detects 5mC [38] (Supplementary Figure 4). As compared to normal tissue, adenocarcinoma of the colon and the prostate only showed a very modest decrease in 5mC intensities (Supplementary Figure 4), and we observed no correlation between 5hmC and 5mC. These data suggest that the global decrease of 5mC cannot alone account for the profound loss of 5hmC levels in solid tumors.

## DISCUSSION

The recent finding that oxidation of 5mC to 5hmC by enzymes of the ten-eleven translocated (TET) family occurs in mammalian genomes has raised many questions regarding the role of this DNA modification in epigenetic regulation. Even though several studies have investigated the complex role of TET proteins and 5hmC in embryonic stem cell biology, the relevance of this mark in developing normal and adult tissues remained essentially unexplored.

Here, we developed a novel, robust immunohistochemical detection method for 5hmC and used this method to detect 5hmC in a large number of murine and human tissues. Interestingly, we found that hierarchically organized epithelia as well as hematopoietic cells in the bone marrow show a differentiation-dependent 5hmC distribution. Cells in the colonic crypt, basal cells of the prostate, as well as hematopoietic stem/progenitor cells exhibited greatly reduced 5hmC levels compared to more differentiated counterparts, suggesting that adult tissue stem/progenitor cells across a broad range of tissue types might be characterized by low 5hmC levels. Differentiation and maturation conversely appeared to be associated with an increase in 5hmC. Based on these data, we can hypothesize that accumulation of 5hmC in the genome is involved in differentiation of tissue stem/progenitor cells. This hypothesis is supported by recent reports showing that genetic disruption of TET2 in hematopoietic cells could lead to increased hematopoietic stem cell self-renewal, accumulation of hematopoietic stem/progenitor cells, and reduced differentiation of hematopoietic stem cells [39, 40].

This observation is somewhat in contrast to recent reports from murine embryonic stem cells, where the differentiation of embryonic stem cells appeared to be associated with a loss in 5hmC [24, 26]. These discrepancies could reflect differences in the biology between embryonic and tissue stem cells and could point to a differential role of 5hmC in very early development versus later development and adult tissue development/differentiation.

Recent reports on the detection of 5hmC in adult tissues have been somewhat conflicting [22, 26]. One explanation for these variable results is certainly the use of different detection methods. In this study, we noted that robust immunohistochemical detection of 5hmC from formalin fixed paraffin embedded tissue requires specific antigen retrieval. Omission of these antigen retrieval steps led to vastly different results (Supplementary Figure 1) and, therefore, explained some of the prevailing discrepancies in the literature.

The functional role of 5hmC in regulating differentiation and epigenetic states of adult tissues remains unknown. It has been proposed that 5hmC cannot be bound by methyl-binding domain proteins such as MeCP2, MBD1, and MBD2 [41-43], which are known to associate with 5mC and recruit the chromatin repression complex. Accumulation of 5hmC could therefore have a significant impact on gene expression states. Moreover, it was suggested that 5hmC is not recognized by the DNA methylation maintenance machinery, suggesting that the presence of 5hmC could lead to a passive loss of DNA methylation during cell division [44]. Most interestingly however, the conversion of 5mC to 5hmC could also represent a mechanism for active demethylation. In a process that involves activation induced deaminase (AID) and base excision repair, 5hmC can be converted to cytosine [45, 46], providing a mechanism for the sequential, active conversion of 5mC to cytosine. Such a process provides an interesting mechanism for plasticity of DNA methylation marks.

Our observation that 5hmC levels are significantly reduced in three different types of human carcinoma suggests that the loss of 5hmC could be a general feature of carcinogenesis. Indeed, in several hematological malignancies including AML and MDS, reduced 5hmC levels have been associated with mutations in the *TET* genes [47, 48]. However, it is unlikely that missense mutation in the TET enzymes can explain the almost universal reduction in 5hmC levels in colorectal, prostate and breast carcinoma, since large scale sequencing efforts have not identified TET family members as frequently mutated in these tumors [49-52]. Recent evidence suggests that a large number of oxidizing enzymes, including the TET family, can be inhibited by oncogenic metabolites, such as 2-hydroxyglutarate [53, 54]. It is, therefore, possible that cancer specific metabolic perturbations can influence 5hmC levels and, consequently, alter the epigenetic makeup of a cell.

In many solid tumors, cancer progression is associated with a progressive loss of 5mC marks resulting in a global hypomethylation phenotype [3, 4]. Since 5mC is required as a substrate for oxidation to generate 5hmC, reduced 5mC levels could explain, at least partly, the decrease of 5hmC observed in tumors. To address a possible correlation between 5hmC and 5mC loss we stained a series of tumor and normal tissues from prostate and colon with an antibody that specifically recognizes 5mC (Supplementary Figure 4). Using this method, we observed only a modest reduction of global 5mC staining intensities between cancerous and normal tissue of the colon and prostate, which is inline with recent reports [38, 55, 56]. Furthermore, we found no association between 5mC and 5hmC staining levels suggesting that the reduction in 5hmC can occur independently of reductions in 5mC.

In conclusion, our study identifies a hierarchical distribution of 5hmC levels in embryonic and adult tissues and provides evidence for a cancer-associated loss of 5hmC.

## MATERIALS AND METHODS

### Sample materials

Mouse embryo tissue was obtained from seventeen-day-old C57BL embryos. All remaining normal adult mouse tissues were from 11 week old FVB mice. All tissues were fixed in 10% buffered formalin immediately after tissue harvest and were embedded into paraffin. Tissue microarrays containing normal and tumor tissue form prostate, breast and colon were constructed at the Johns Hopkins TMA core facility.

### Pathological evaluation

Samples were assessed by using an H-score system obtained by multiplying the intensity of the stain (0: no staining; 1: weak staining; 2: moderate staining; 3: intense staining) by the percentage (0 to 100) of cells showing that staining intensity (H-score range, 0 to 300). Only nuclear staining in epithelial cells was evaluated, either in tumor or benign tissues. Since nuclear 5hmC staining was robustly detected in stromal cells associated with tumor or benign tissue, only samples with strong stromal staining were evaluated as a means of censoring tissue samples that did not stain for 5hmC due to fixation or other artifacts.

### Immuno-labeling of 5hmC and 5mC

To generate positive controls for 5hmC staining optimization, HEK293 cells were transiently transfected with myc-tagged TET2 constructs (obtained from Dr. Ari Melnick [53]) or vector controls using Lipofectamine 2000 (Invitrogen, Carlsbad, CA). Cell pellets were fixed in 10% buffered formalin and embedded in paraffin as described previously [23]. 5 micron paraffin sections were de-waxed and rehydrated following standard protocols. Antigen retrieval consisted of steaming for 30 min in citrate buffer (pH 6.0) followed by incubation in 3.5 N HCl for 15 min at room temperature. Slides were washed and equilibrated in TBS-Tween buffer (Sigma, St. Louis, MO) for 10 min. The same antigen retrieval was used for 5mC and 5hmC. For immunolabeling of 5hmC, the rabbit polyclonal 5 hydroxymethylcytosine specific antibody (Active Motif, Cat # 39769, Carlsbad, CA) was applied at 1:20,000 dilution. For 5mC detection, the mouse monoclonal 5 methylcytosine specific antibody (Calbiochem, EMD Chemicals Inc., San Diego, CA) was used at 1:2000 dilution. Both primary antibodies were incubated for 1 h at room temperature. Immuno-complexes were detected using the the PowerVision + ™ immunohistochemistry detection system from ImmunoVision Technologies Co (Norwell, MA, USA) with 3,3'-diaminobenzidine tetrahydrochloride (DAB) as the chromogen. After immunohistochemical staining, tissue sections were counterstained with hematoxylin.

For immunofluorescence analysis, slides were pretreated as outlined above and incubated with rabbit polyclonal 5hmC specific antibody (Active Motif, Cat # 39769) at a 1:8000 dilution with or without mouse monoclonal antibodies specific to myc (9E11, Santa Cruz, CA), cytokeratin 34βE12-903 (ENZO, Farmingdale, NY) or cytokeratin 15 (Ab-1, NeoMarkers, Fremont, CA ) at 1:50 dilutions. Immuno-complexes were further labeled with secondary antibodies conjugated with Alexa 488 or Alexa 568 dyes (Invitrogen) and DNA was counterstained with DAPI. Slides were then visualized using a Nikon E400 fluorescence microscope (Nikon Instruments, Melville, NY).

To quantitate 5hmC levels in different cell compartments, representative images of 5hmC and 903 or CK15 co-labeled slides were analyzed using the Telometer software application [57]. Therefore, signal intensities of individual cell nuclei in the basal and luminal/apical cell compartment were determined. To account for differences in overall DNA content, 5hmC signal intensities were normalized to DAPI intensities.

### Isolation of hematopoietic cells

Hematopoietic stem and progenitor cells were isolated as described previously [32]. In brief, bone marrow samples were obtained from healthy individuals and mononuclear cells were isolated from fresh samples by Ficoll-Paque density centrifugation. To enrich for CD34 positive cell populations, cells were selected by Miltenyi Biotec columns (Auburn, CA). CD34 negative cells were spotted on glass slides. Aldehyde Dehydrogenase (ALDH) activity was assessed in CD34 positive cells by staining with Aldefluor (Aldagen, Durham, DC). Cells were further immuno-labeled with anti-CD34 and anti-CD38 antibodies, sorted into CD34^+^CD38−ALDH^high^ and CD34^+^CD38^+^fractions and directly spotted on microscope slides. Samples were then stained with 5hmC specific antibodies.

## Supplementary Figures


