# Supplementary material for: Global 5-hydroxymethylcytosine content is significantly reduced in tissue stem/progenitor cell compartments and in human cancers
Source: Oncotarget. 2011 Sep 2;2(8):627–37. doi: 10.18632/oncotarget.316 (PMC3248214; doi:10.18632/oncotarget.316)
Supplement: Supplementary file 1 [file oncotarget-02-627-s001.pdf]

Supplementary Figures

Global 5-hydroxymethylcytosine content is significantly reduced in tissue stem/progenitor cells compartments and in human cancer

Michael C. Haffner, Alcides Chaux, Alan K. Meeker, David M. Esopi, Jonathan Gerber, Laxmi G. Pellakuru, Antoun Toubaji, Pedram Argani, Christine Iacobuzio-Donahue, William G. Nelson, George J. Netto, Angelo M. De Marzo, Srinivasan Yegnasubramanian

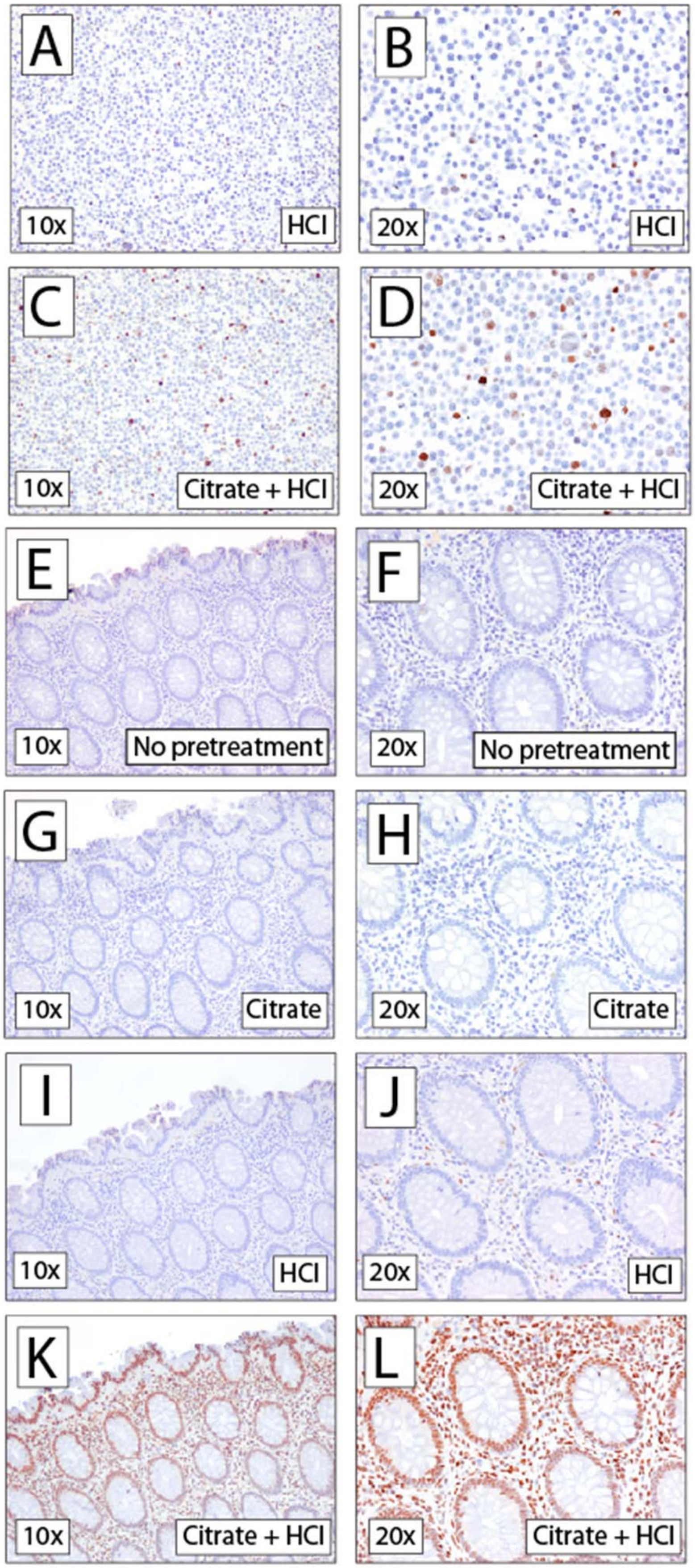

Supplementary Figure 1.
Adequate antigen retrieval is necessary to obtain robust immuno-labeling of 5hmC in formalin fixed paraffin embedded tissue sections.
HEK293 cells transiently transfected with TET2 were fixed in formalin and embedded in paraffin. Tissue sections were either pretreated in 3.5 N HCl for 15 min alone (A, B) or steamed in citrate buffer (ph 6) for 30 min and then incubated in HCl (C, D). Slides were then stained with 5hmC specific antibodies and immunoreactive complexes were visualized with DAB. Note that pretreatment with HCl alone showed only faint staining, whereas the combination pretreatment of citrate steaming and HCl incubation showed robust labeling of 5hmC. Similarly, formalin fixed paraffin embedded sections of normal colon mucosa exhibited no 5hmC staining (E, F) when all pretreatment steps were omitted. Likewise, citrate steaming (G, H) or HCl treatment (I, J) alone did not result in 5hmC labeling. However, a combination of citrate steaming and HCl pretreatment resulted in efficient immunolabeling of 5hmC in normal human colon mucosa.

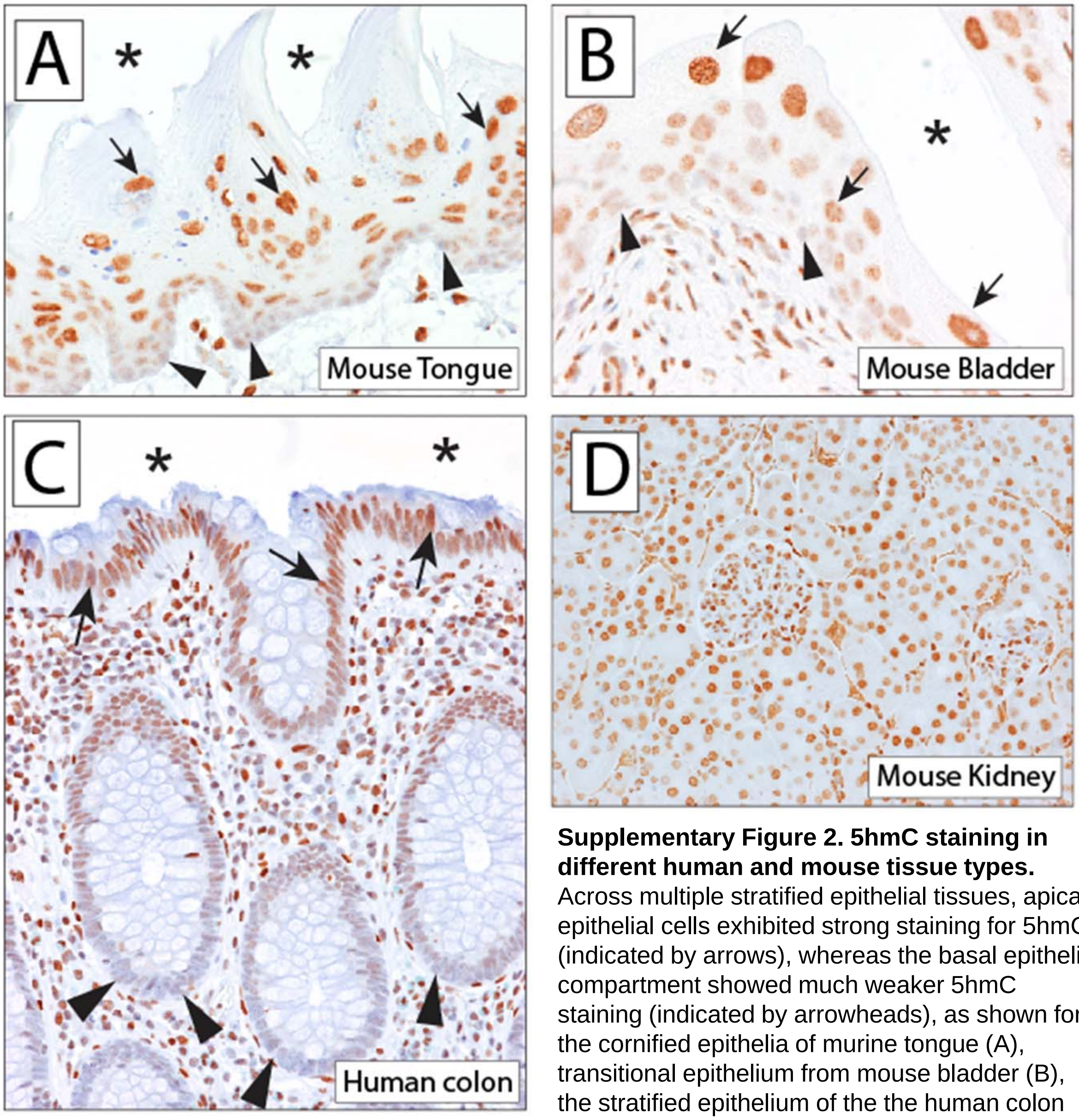

Supplementary Figure 2. 5hmC staining in different human and mouse tissue types. Across multiple stratified epithelial tissues, apical epithelial cells exhibited strong staining for 5hmC (indicated by arrows), whereas the basal epithelial compartment showed much weaker 5hmC staining (indicated by arrowheads), as shown for the cornified epithelia of murine tongue (A), transitional epithelium from mouse bladder (B), the stratified epithelium of the the human colon (C). Murine kidney (D) tissue showed strong immunoreactivity for 5hmC in the majority of cell nuclei. Asterisks (*) indciate the location of the lumen.

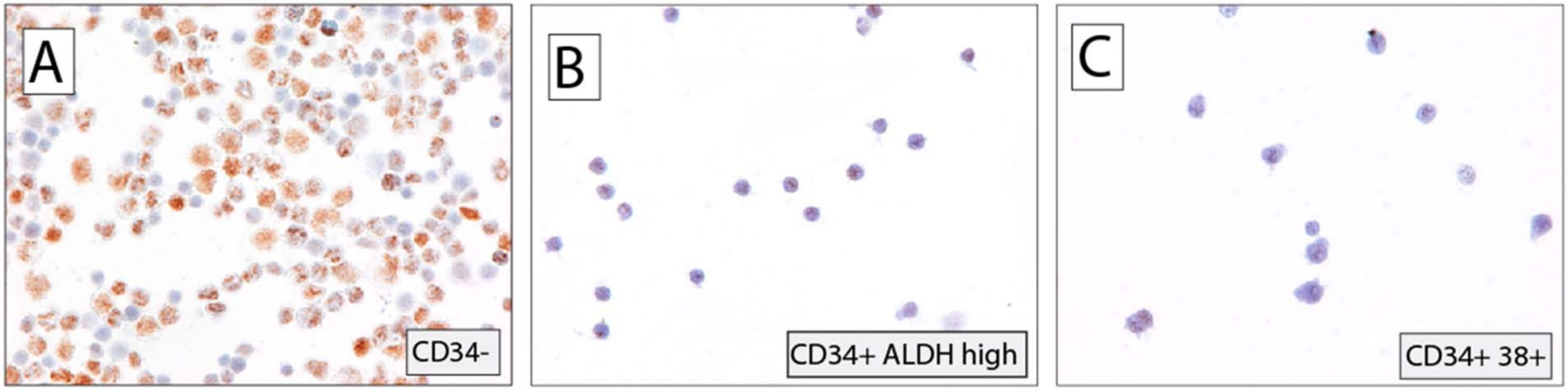

Supplementary Figure 3. 5hmC levels are low in hematopoietic stem/progenitor cells.
5hmC immunohistochemical staining in (A) Ficoll-Paque enriched, CD34-depleted bone marrow, (B) $CD34^+CD38^-ALDH^{high}$ hematopoietic stem cells and (C) $CD34^+CD38^+$ progenitor cells. Bone Marrow samples were obtained from healthy individuals and mononuclear cells were isolated from fresh samples by Ficoll-Paque density centrifugation. To enrich for $CD34^+$ cell populations, cells were selected by Miltenyi Biotec columns. ALDH1A1 activity was assessed in $CD34^+$ cells by staining with Aldefluor. Cells were further immunolabled with anti-CD34 and anti-CD38, sorted into $CD34^+CD38^-ALDH^{high}$ and $CD34^+CD38^+$ fractions and directly spotted on microscope slides. Samples were then stained with 5hmC specific antibodies. Representative micrographs of all 3 fractions are shown. Note that 5hmC staining is greatly reduced in the $CD34^+CD38^-ALDH^{high}$ (B) and $CD34^+CD38^+$ (C) cell populations.

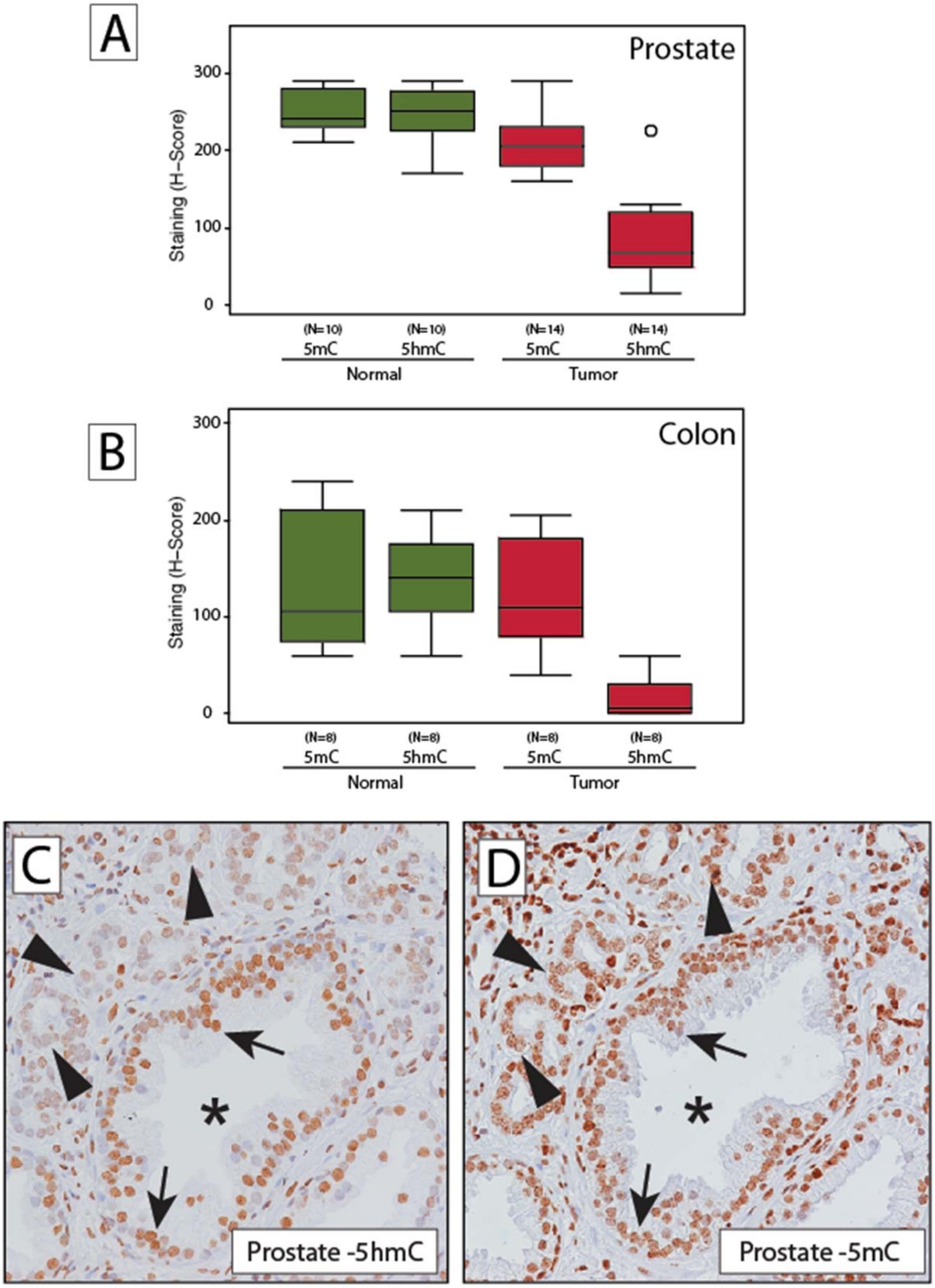

Supplementary Figure 4. Decrease of 5hmC is independent of 5mC.
To assess if loss of 5hmC in cancers is accompanied by decreased 5mC levels, normal and tumor tissue of prostate (A) and colon (B) were stained with 5hmC and 5mC specific antibodies and staining intensities and distribution of 5mC and 5hmC was assessed semiquantitatively using the H-score system. Whereas 5hmC levels were profoundly reduced in prostate and colon adenocarcinoma, global 5mC levels were only modestly decreased. No correlation between 5hmC and 5mC staining was observed. (C, D) Representative micrographs of adjacent sections showing greatly decreased 5hmC staining in prostate cancer cells (C, arrowheads) but no reduction in 5mC staining (D). Note that normal luminal cells (arrows) show strong staining for 5hmC and 5mC.
